# Influence of EDM Process Parameters on the Surface Finish of Alnico Alloys

**DOI:** 10.3390/ma15207277

**Published:** 2022-10-18

**Authors:** Damian Bańkowski, Piotr Młynarczyk

**Affiliations:** Department of Metal Science and Manufacturing Processes, Faculty of Mechatronics and Mechanical Engineering, Kielce University of Technology, al. Tysiąclecia Państwa Polskiego 7, 25-314 Kielce, Poland

**Keywords:** electrical discharge machining (EDM), alnico, surface finish, roughness, surface quality

## Abstract

This article deals with electrical discharge machining (EDM) of an alnico alloy, focusing on how key process parameters affect the surface finish. The experiments were conducted using a BP93L EDM machine. The Box–Behnken design was employed to study the effects of three factors, i.e., spark current, pulse-on time, and pulse-off time, each at three levels, on the surface quality. A specially designed system was employed to increase the effectiveness of the machining process by imparting an additional rotary motion to the tool and an additional rotary motion to the workpiece. The aim was to efficiently remove the eroded metal particles and create a surface with smaller craters. The workpiece surface roughness was measured with a Talysurf CCI lite non-contact profiler. During this precision machining process, the arithmetical mean height (*Sa*) was less than 1 µm. The surface quality was examined also using scanning electron microscopy (SEM) and optical microscopy (OM). The experimental data were analyzed by means of Statistica to determine and graphically represent the relationships between the input and output parameters.

## 1. Introduction

The surface generated in electrical discharge machining is a result of thermal phenomena evoked by electrical discharges in the gap between the tool and the workpiece. Surface texture analysis provides information on how the surface formation is dependent on the machining conditions [1,2,3,4,5]. Surface texture measurement and analysis are particularly important for electrical discharge machining or other unconventional machining processes, which have not been investigated thoroughly because of the complexity of physical processes and their interactions [6,7,8,9].

In EDM, material is removed by rapidly recurring discharges between the tool and the workpiece. The process is stochastic in nature because the sparking takes place between points of the lowest electrical resistance [10]. Typically, the EDM process requires the workpiece and the tool to be submerged in a dielectric fluid so that stable (thermal) conditions are maintained for the melting or vaporization of a microscopic bit of the workpiece [11]. The EDM fluid also acts as a flushing agent, and helps effective clean out of debris created during cutting [12]. EDM is generally used to produce geometrically complex parts. The required geometry of the workpiece is achieved by using a tool replicating this geometry in negative. The tool and the workpiece are not in contact during machining as the gap between them is filled with a dielectric fluid. It is assumed that there are no mechanical interactions between them influencing the process or the workpiece deformations.

The surface resulting from EDM is characterized by overlapping craters. Their geometric features, including radius, height, depth, curvature, and spacing, determine the surface texture [13,14,15,16,17], which can be controlled by applying appropriate process parameters (voltage, spark current, pulse-on time, etc.), material and shape of the electrode, and type of dielectric fluid [18,19]. The crater size depends on the energy of a single discharge. Klocke et al. claim that the depth of a recast layer is attributable to the resistance and capacity of the charging circuit, as both are responsible for the discharge energy, and higher energy results in a thicker recast layer [20]. Giridharan et al. propose the so-called anode model, which assumes that the energy reaching the workpiece is the key factor in crater formation and that the crater diameter is proportional to the discharge energy [21]. Other studies indicate proportional relationships between discharge energy and crater volume [22], area [23], diameter [24] or size [25]. Ding et al. showed that in microwire electrical discharge machining, the spark energy has a direct influence on both the average diameter and the maximum depth of craters. Masuzawa et al. indicated that when low open-circuit voltage is applied, smaller craters form and the resulting surface roughness (*Ra*) is lower [26]. Guu points out that higher spark currents and longer pulse-on times are responsible for higher values of the parameter *Ra* [27]. From a review of the literature, it is evident that there are strong relationships between the geometric dimensions of craters and the discharge energy. The crater size is also dependent on the electrode material, workpiece material and type of EDM fluid [28,29].

Many studies on EDM have considered the influence of various factors on the process efficiency and effectiveness [30,31]. Only some, however, have dealt with changes in the material surface texture. Arooj et al. [32], for example, analyzed how electric current affects the surface morphology of the workpiece. They showedthat at higher current density, the process takes place more rapidly, but the surface roughness increases from 2.5 to 4.5 μm. Much of the research in this area focuses on the machining of iron alloys [31,33,34,35,36] and aluminum alloys [30,31,37,38,39,40]. The effects of the EDM parameters on nickel microstructure are described by Bai et al. [41]. Up till now, there have been no other team studies on the behavior of alnico alloys (iron-based alloys containing aluminum, nickel, and cobalt) when machined by EDM. This article investigates the effects of the selected process conditions on the changes in the surface texture of the material.

Alnico alloys were selected for this study because of their mechanical properties, particularly high brittleness and relatively high hardness. In such a case, conventional machining, especially grinding, is not possible. The resulting surface may feature chipping, surface burns, pull-outs, and microcracking. Electrical discharge machining is thus proposed as an alternative solution to surface shaping. Electrical discharge machining is a non-contact process with no mechanical interactions between the tool and the workpiece and therefore limited vibration, which ensures higher geometric accuracy and a better surface finish.

A review of the literature shows that there is hardly any information about EDM of permanent magnet materials. From a physical point of view, the process is difficult or even impossible to carry out. The tool may get stuck in the workpiece if the spark-gap is short-circuited by the debris. The motivation behind the research was to replace conventional machining of alnico alloys with unconventional machining, i.e., EDM. Alnico alloys are brittle materials, anddamage encountered in traditional machining includes cracking and edge chipping. Experiments have shownthat it was essential to apply additional relative motion between the tool and the workpiece. An additional motion of the tool relative to the workpiece wasanalyzed, for instance, in [42]. The research results indicate that in such a case debris removal is easier, and the process is faster [42].

The purpose of this article is to show that the surface of alnico alloys machined by EDM can be controlled by modifying the input process parameters. The research involved determining the mathematical relationships between the process parameters (spark current, pulse-on time, and pulse-off time) and the quality of the surface, characterized by area field (3D) roughness parameters.

According to PN-EN ISO 25178-6:2011 [43], surface texture can be studied using linear profiling (2D roughness) and methods of spatial topography (3D roughness). Generally, 3D roughness parameters are determined. Their measurement can be done using portable instruments or special-purpose industrial measurement systems. However, data obtained by linear profiling is not sufficient to fully characterize surface quality [44,45]. For new or modified machining processes, a complex analysis based on 3D surface roughness data is recommended. There are two main groups of methods that can be used to measure surface topography: contact and non-contact (optical). Although the latter are faster, they may not be suitable for certain types of surfaces. Contact methods, considered to be more accurate, are generally used in industrial conditions.

## 2. Materials and Methods

Experiments were carried out to study the effects of EDM on alnico alloys. Alnico is the name of a family of iron-based alloys, which, as the name suggests, contain aluminum, nickel, and cobalt (Al-Ni-Co). The chemical composition is generally 7–10% Al, 13–16% Ni, and 20–40% Co, with the rest being Fe [46]. In the case of alnico magnets, copper (3–5%), titanium (1–8%), Nb, Ta, and other elements may also be added. Alnico alloys are produced metallurgically either by casting or sintering (powder metallurgy) [46]. There are a total of 29 grades of alnico alloys: 17 grades of cast alloys, 10 grades of sintered alloys, and 2 bonded grades. The various grades are known by the trade names Alnico, Columax, Alcomax 3SC, Alni, Hycomax, and Ticonal [47]. The Alnico alloy tested was of the cast type. The composition of the material, determined by a JEOL JSM-7100F field emission scanning electron microscope, is shown in Table 1.

Alnico alloys are ferromagnetic. They also have high coercivity, i.e., high resistance to demagnetization, which makes them suitable for permanent magnets. On their introduction in the 1930s, this property made alnico magnets superior to ubiquitous electromagnets. Alnico alloys are well suited for magnets because of their ability to produce strong magnetic fields. Magnetic fields at their poles can reach 1500 gauss, or 0.15 tesla; they are approximately 3000 stronger than the Earth’s magnetic field. These days, alnico magnets are outperformed only by magnets made from rare earth elements, i.e., neodymium magnets and samarium–cobalt magnets. Alnico magnets are also characterized by high resistance to corrosion, extremely high Curie point, Tc, and impressive magnetic stability over a large range of temperatures, with working temperatures reaching 525 °C [46]. Some alnico magnets are isotropic, i.e., with the same properties in all directions, and their magnetic fields are strong in all directions. Other alnico magnets are anisotropic, which means their properties differ depending on direction. Such magnets can only be magnetized in one preferred direction. Examples of anisotropic alnico alloys include alnico 5 and alnico 8 [47]. It is important to note, however, that anisotropic alnico alloys with a preferred magnetization direction make stronger magnets than isotropic alnico alloys.

Alnico magnets are primarily used in measuring and control devices, including transducers and sensors, motors and generators, and many other systems where a stable magnetic field is necessary and whose structure allows such a magnet to be installed.

**Figure 1:**
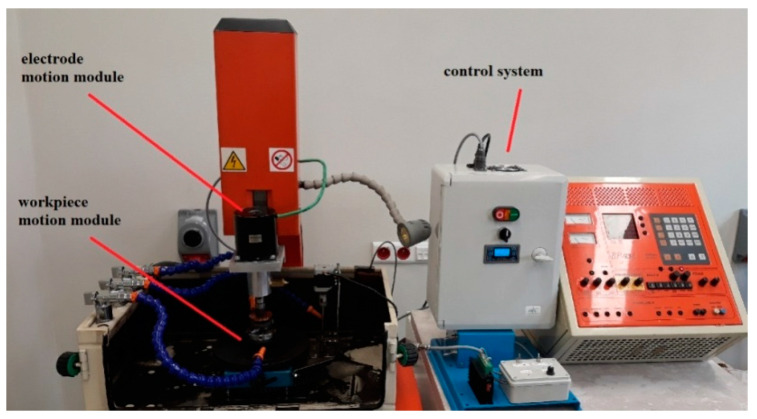
A system providing additional rotational motions to the tool and the workpiece.

The experiments were carried out using a 3 kW BP93L EDM machine with a maximum spark current of 50 A. Kerosene was used as the dielectric fluid. Forced circulation filtration was required to ensure its dielectric stability. The tests were conducted using thespecially developed system illustrated in Figure 1. The additional relative motion between the tool and the workpiece helped remove the eroded metal particles from the spark-gap more efficiently. This system makes it possible to fully control the tool and workpiece drives, i.e., to change the direction and speed of their rotations. Because of this rotational motion, the surface finish is more uniform.

During the first EDM tests, when no additional rotational motion was used, the process lasted no longer than 30−60 s. As a permanent magnet material was cut, short circuits occurred, and the debris could not be removed from the cutting zone. It was necessary to retract the tool and clean out the eroded material from the spark gap. When an additional rotational motion is used to machine permanent magnets, the process is shorter because the debris removal is faster [42]. The tests described in this article showed that the machining time could be reduced by applying an additional rotational motion. As a result, a smaller amount of eroded material was produced, and this contributed to lower surface roughness.

The Box–Behnken three-factor, three-level design of experiments was applied to determine the effects of the process parameters on the surface finish parameters. The three key factors affecting the EDM process were selected on the basis of a literature review [48] and preliminary research. In the study, the input parameters were spark current *I*, pulse-on time *t_on_*, and pulse-off time *t_off_*. Three different EDM parameters (current *I*, pulse-on time *t_on_*, and pulse-off time *t_off_*) were considered using a three-level Box–Behnken design with three variables, as shown in Table 2.

The 3D surface roughness parameters were measured using a Taylor-Hobson Talysurf CCI Lite non-contact 3D profiler. The standard number of measurement points was 1024 × 1024, and the resolution (X−Y) at 50× magnification was 1.33 μm. Microstructural examinations were carried out by means of a Nikon Eclipse MA200 optical microscope equipped with NIS 4.20-Elements Viewer imaging software. The mathematical relationships between the input and output parameters were visualized using Statistica 10.

## 3. Results

**Figure 2:**
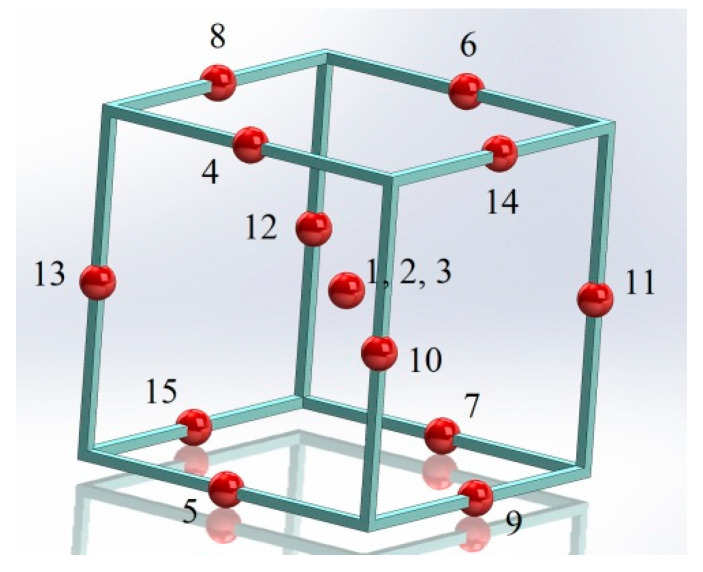
The three-factor, three-level design of experiment DOE.

Performing experiments for many different settings of the input parameters would have been very time consuming. The Box–Behnken design was employed to reduce the time required for the tests by reducing the number of experiments from 27 to 15. The design points to be used in the experiments are shown in Figure 2.

Combinations of the process conditions generated by Statistica are shown in Table 3. Table 3 also provides the corresponding measurement data concerning the surface quality.

The parameter *Sa* is the arithmetical mean height of a line to the surface, *Sp* is the maximum peak height within the defined area, and *Sv* is the maximum pit height within the defined area. *Sz*, the maximum height, is the sum of *Sv* and *Sp*.

It should be noted that the parameters *Sa* and *Sz* cannot be used to characterize surface roughness, andit is vital to employ the functional parameters to describe the particular irregularities. The parameter *Ssk*, skewness, was also analyzed.

**Figure 3:**
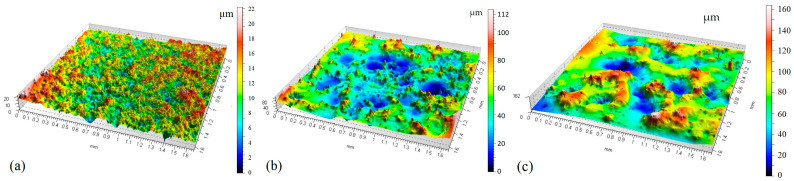
3D isometric views of the workpiece surface after EDM. Process parameters: *t_on_* = 200 µs, *t_off_* = 10 µs, and (**a**) *I* = 5 A; (**b**) *I* = 10 A; (**c**) *I* = 15 A.

Figure 3 shows three-dimensional images of the alnico alloy surface produced by electrical discharge machining at a pulse-on time of 200 µs, a pulse-off time of 10 µs, and a spark current of 5, 10 or 15 A.

Analysis of the 3D images of the alnico alloy machined by EDM (Figure 3) revealedthe effects of electrical discharges with different energies. For a spark current of 5 A (Figure 3a), the surface finish was uniform with a large number of overlapping craters. The parameter *Sa* was 2.0 µm. In the other case (depicted in Figure 3c), *Sa* reached 20 µm; wider and deeper craters were observed. The number of roughness peaks and valleys washigher at 15 A than at 5 A and 10 A. From Figure 3, it is also clear that the surface finish obtained at 5 A (Figure 3a) wasmore uniform than that produced at 10 A (Figure 3b) or 15 A (Figure 3c). When a higher spark current of 15 A was applied, there were much fewer sharp peaks, but they were higher. At a lower spark current of 5 A, the number of peaks increased and their height above the reference height was about 10 times lower.

**Figure 4:**
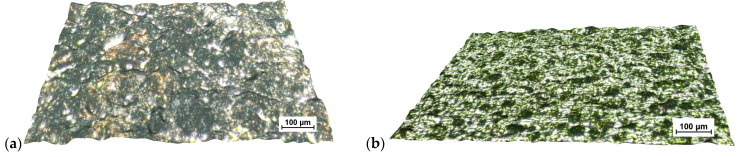
Micro images of the surface after EDM for: (**a**) high spark energy; (**b**) low spark energy.

The results of the OM examinations shown in Figure 4 confirm the stereometric features.

**Figure 5:**
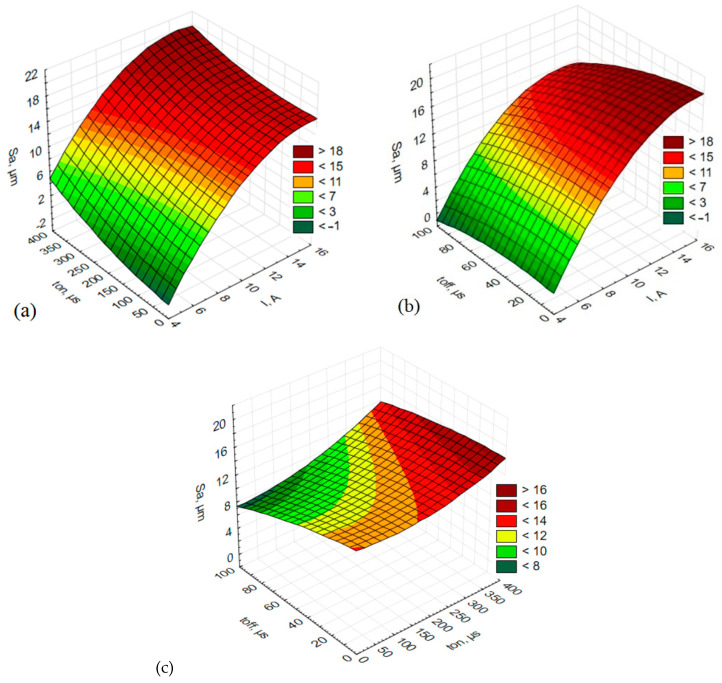
The estimated response surface plot of arithmetical mean height (*Sa*): (**a**) constant *t_off_* = 50 µs; (**b**) constant *t_on_* = 200 µs; (**c**) constant *I* = 15 A.

The parameter *Sa* plotted as a function of different combinations of the input parameters is illustrated in Figure 5.

From the diagrams in Figure 5, it is evident that the arithmetical mean height increases with increasing spark current when both the pulse-off time and pulse-on time are taken into account (Figure 5a,b), respectively). The analysis of the effects of the pulse-on time and pulse-off time reveals that the lowest values of the arithmetical mean height, *Sa*, can be obtained at a short pulse-on time and a long pulse-off time (Figure 5c). An increase in the pulse-on time and a decrease in pulse-off time leads to an increase in the parameter *Sa*.

From Table 3, illustrating the height parameters, i.e., the maximum peak height and the maximum pit height (*Sp* and *Sv*, respectively), it is clear that the values of *Sp* are generally higher than those of *Sv*. This suggests more numerous high peaks than pits, because crater formation occurs during a microdischarge. The crater bottom resembles a spherical cap, whereas the crater rim is a result of rapid melting of liquid metal and, by nature, is irregular in shape.

**Figure 6:**
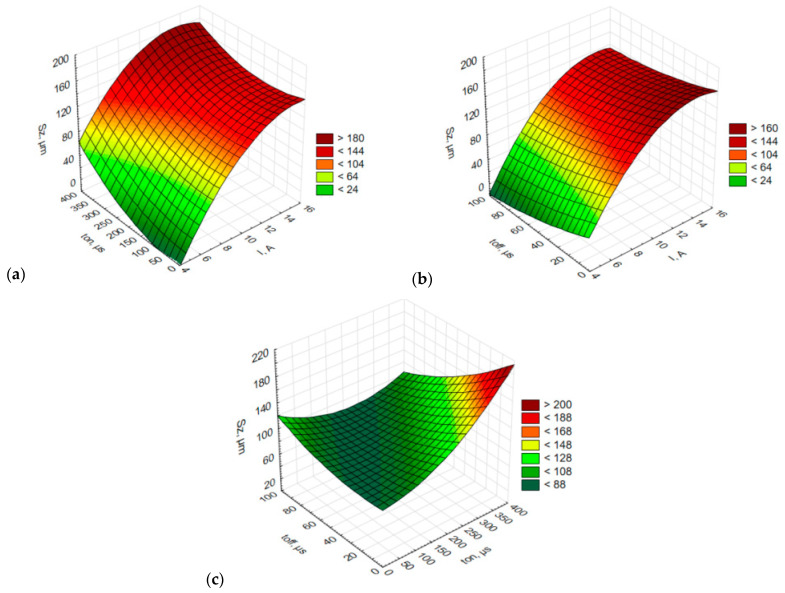
Estimated response surface plot of the maximum height (*Sz*): (**a**) constant *t_off_* = 50 µs; (**b**) constant *t_on_* = 200 µs; (**c**) constant *I* = 15 A.

Since *Sz* is the sum of *Sp* and *Sv*, *Sz* was used to study the relationships between the process parameters and the surface roughness. The conclusions concerning the maximum height, *Sz*, drawn from the analysis of the plots in Figure 6, are similar to those obtained for *Sa*.

**Figure 7:**
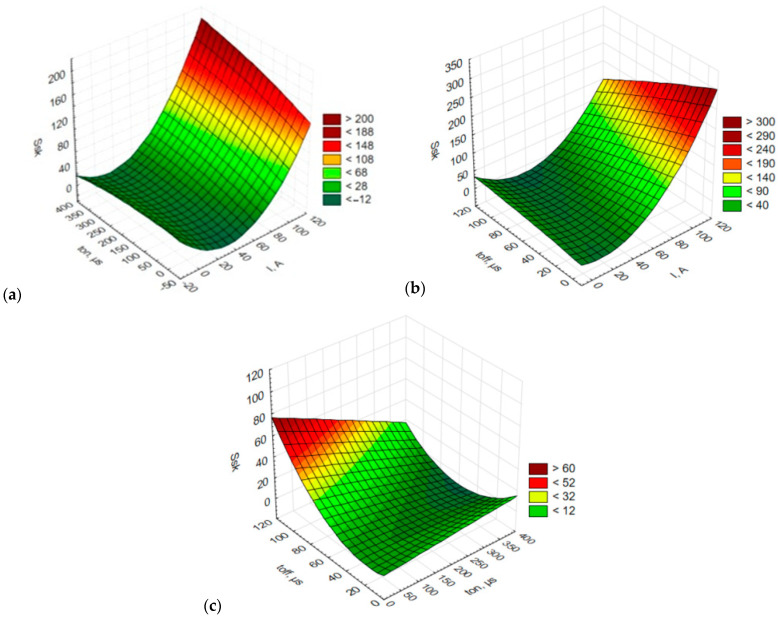
Estimated response surface plot of skewness (*Ssk*):(**a**) constant *t_off_* = 50 µs; (**b**) constant *t_on_* = 200 µs; (**c**) constant *I* = 15 A.

Figure 7 depicts changes in the skewness (*Ssk*) depending on the input EDM parameters.

As can be seen from Figure 7, skewness was affected mainly by changes in the spark current. An increase in spark current in EDM is responsible for an increase in skewness, ascan be observed in Figure 7a,b. From the diagram in Figure 7c it is evident that skewness is more dependent on the pulse-off time, *t_off_*, than on the pulse-on time, *t_on_*. At longer pulse-off times, the values of skewness, *Ssk*, are higher. Analysis of the values of *Ssk* revealed that the parameter fluctuated around zero, which suggests that the distribution of roughness wassimilar to the normal distribution. When *Ssk* was greater than zero, the surface was characterized by sharper peaks and, therefore, a greater concentration of materials in the valleys. This observation is important when finishing or coating operations are required. A material with such a morphology is easier to machine conventionally as the removal of fewer peaks is less troublesome and a smoother surface is not difficult to obtain.

The conclusions drawn for the amplitude parameters *Sa* and *Sz* are similar to those obtained from the analysis of skewness (*Ssk*); in both cases, positive values were generally obtained. The data indicate that the higher the spark current used to machine the alnico alloy tested, the more asymmetric the surface texture. The asymmetry increasedrapidly with increasing pulse-on time (Figure 7a) and decreasing pulse-off time (Figure 7b).

A coefficient of discharge, αt, was introduced to find the correlation between the pulse-on time and the pulse-off time in EDM (Equation (1)):

(1) $$\alpha_{t}=\frac{t_{on}}{t_{on}+t_{off}}$$

where: *α_t_*—coefficient of discharge, *t_on_*—pulse-on time, and *t_off_*—pulse-off time.

The mathematical models developed on the basis of the experimental data describe the effects of the process parameters on the surface texture. The regression equations were derived for a second-degree polynomial function (Table 4). The calculations were made at the 95% level of confidence.

The correlation coefficient R, describing the variability of the feature, was also determined. Table 5 shows the correlation between the process parameters and the spatial surface texture parameters.

There were very strong correlations between the spark current and almost all the surface texture parameters, except for *Sa*, *Sp*, *Sv*, *Sz* and *Ssk*. Similar observations were made for the correlations between the pulse-on time and the surface texture parameters; however, those for *Sa*, *Sp*, *Sv*, *Sz*, were low, while those for *Ssk* were negligible. The correlations concerning the pulse-off time were either negative or poor for all the output parameters. A negative moderate correlation wasobtained at higher pulse-off times. It should be noted that skewness, *Ssk*, was dependent on both the pulse-off-time and the spark-current.

In EDM, changes in the spark current are mainly responsible for changes in the spatial parameters (*Sa*, *Sp*, *Sv*, *Sz*). At lower values of the spark current, *I*, the erosion of particles is slower. The debris is removed in very small amounts by evaporation. The higher the spark current, the higher the power of a single discharge and, consequently, the greater the erosion of the material particles (larger vapor drops), especially when the pulse-on time is longer. From a physical point of view, longer pulse-on times, *t_on_*, mean longer discharge times and longer plasma channel formation times. Under such conditions, large, deep valleys form. Short discharge times contribute to deeper but smaller valleys. Longer pulse-off times result in lower values of the spatial parameters, andthe flushing of the debris from the cutting zone is more efficient. Shorter pulse-off times, *t_off_*, lead to inefficient removal of solid particles, which may cause short circuits and surface damage.

From Table 4, it is apparent that there is a positive poor correlation between longer pulse-on times and the spatial and frequency parameters. The opposite conclusions were drawn while analyzing the pulse-off times. Longer times between discharges led to lower surface roughness, i.e., lower values of the spatial and frequency parameters.

The lowest surface roughness was observed at very low spark current. However, small energies of single discharges were responsible for lower effectiveness of the processes. The removal of the eroded material took much longer. It is, thus, recommended that the electrical discharge machining process should be performed in two or even three stages. The first stage requires high power (high spark current, I, and high voltage, U), long pulse-on times, and short pulse-off times. The spark current and the time parameters are reduced gradually with every next step, depending on the surface quality requirements.

## 4. Conclusions

The study results show that the spark current has a crucial role in the electrical discharge machining of alnico alloys, and is the key factor affecting surface quality. An increase in spark current caused an increase in amplitude and spatial parameters. An appropriately short pulse-on time and an appropriately long pulse-off time contributed to lower values of the 3D surface texture parameters. The application of an additional rotary motion to both the workpiece and the tool helped improve the effectiveness of the EDM process, i.e., to reduce the surface roughness and better remove the debris from the spark-gap.

(1) The higher the spark current, *I,* the higher the values of the spatial parameters (*Sa*, *Sp*, *Sv*, *Sz*) and the frequency parameter (*Ssk*).
(2) The values of the spatial parameters (*Sa*, *Sp*, *Sv*, *Sz*) increase with increasing pulse-on time. The pulse-on time, however, has a negligible effect on the frequency parameter (*Ssk*).
(3) Longer pulse-off times result in lower values of both the spatial and frequency parameters.
(4) The lowest arithmetical mean height, *Sa* = 0.281 µm, was obtained at *I* = 5 A, *t_on_* = 50 µs, and *t_off_* = 50 µs. The highest value of *Sa*, i.e., 20.8 µm, was reported for *I* = 15 A, *t_on_* = 350 µs, and *t_off_* = 50 µs.

## Figures and Tables

**Table 1:** Chemical composition of the Alnico alloy tested (wt.%).

Element	Al	Ti	Fe	Co	Ni	Cu
Average (wt.%)	17.9	0.9	35.1	26.2	15.3	4.6

**Table 2:** Box–Behnken experimental design: the code values and the corresponding actual (experimental) values of the process parameters.

Number of Experiment	Code Values			Actual Values—Input		
	*I*	*t_on_*	*t_off_*	*I*, A	*t_on_*, µs	*t_off_*, µs
1	0	0	0	15	200	50
2	0	0	0	15	200	50
3	0	0	0	15	200	50
4	0	1	1	15	350	90
5	0	1	−1	15	350	10
6	0	−1	1	15	50	90
7	0	−1	−1	15	50	10
8	1	0	1	25	200	90
9	1	0	−1	25	200	10
10	1	1	0	25	350	50
11	1	−1	0	25	50	50
12	−1	−1	0	5	50	50
13	−1	1	0	5	350	50
14	−1	0	1	5	200	90
15	−1	0	−1	5	200	50

**Table 3:** The input and output parameters for the 15 tests.

Input Parameters								
No.	*I*, A	*t_on_*, µs	*t_off_*, µs	*Sa*, µm	*Sp*, µm	*Sv*, µm	*Sz*, µm	*Ssk*
1	10	200	50	13.4	70.4	43.3	114	0.355
2	10	200	50	15.4	68.7	47.4	116	0.368
3	10	200	50	12.6	60.7	46.9	108	0.342
4	10	350	90	11.2	51.4	38.0	89.4	0.27
5	10	350	10	13.7	119.0	67.0	186	0.674
6	10	50	90	12.8	68.9	49.3	118	0.219
7	10	50	10	17.3	81.0	48	129	0.432
8	5	200	90	0.467	1.65	4.41	6.06	−1.95
9	15	200	10	20.6	89.3	70.0	159	0.300
10	15	350	50	20.8	89.2	74.5	164	0.029
11	15	50	50	12.0	65.8	49.5	115	0.353
12	5	50	50	0.281	0.90	0.8	1.74	0.067
13	5	350	50	11.2	51.4	38.0	89.4	0.270
14	15	200	90	16.1	98.0	57.1	155	0.468
15	5	200	10	2.29	10.8	11.9	22.7	0.245

**Table 4:** Regression equations describing the surface texture.

$Sa=-9.20+3.41\cdot I-16.76\cdot \alpha_{t}-0.13\cdot I^{2}+0.65\cdot I\cdot \alpha_{t}+15.06\cdot \alpha_{t}{}^{2}$
$Sp=-54.18+26.44\cdot I-202.1\cdot \alpha_{t}-0.9\cdot I^{2}-2.13\cdot I\cdot \alpha_{t}+201.8\cdot \alpha_{t}{}^{2}$
$Sv=1.92+12.44\cdot I-177.85\cdot \alpha_{t}-0.38\cdot I^{2}+0.11\cdot I\cdot \alpha_{t}+155\cdot \alpha_{t}{}^{2}$
$Sz=-53.42+38.92\cdot I-376.94\cdot \alpha_{t}-1.28\cdot I^{2}-1.96\cdot I\cdot \alpha_{t}+354.47\cdot \alpha_{t}{}^{2}$
$Ssk=-1.43+0.53\cdot I-4.84\cdot \alpha_{t}-0.02\cdot I^{2}-0.20\cdot I\cdot \alpha_{t}+5.45\cdot \alpha_{t}{}^{2}$

**Table 5:** Surface texture parameters correlated with the input factors in EDM.

	*I*, A	*t_on_*, µs	*t_off_*, µs
*Sa*, µm	0.81	0.21	−0.20
*Sp*, µm	0.76	0.26	−0.22
*Sv*, µm	0.83	0.30	−0.20
*Sz*, µm	0.80	0.28	−0.22
*Ssk*	0.39	0.03	−0.41

## Data Availability

No publicly archived datasets are reported or were used.
